# Albumin-Associated Lipids Regulate Human Embryonic Stem Cell Self-Renewal

**DOI:** 10.1371/journal.pone.0001384

**Published:** 2008-01-02

**Authors:** Francesc R. Garcia-Gonzalo, Juan Carlos Izpisúa Belmonte

**Affiliations:** 1 Gene Expression Laboratory, The Salk Institute for Biological Studies, La Jolla, California, United States of America; 2 Center of Regenerative Medicine in Barcelona, Barcelona, Spain; Fred Hutchinson Cancer Research Center, United States of America

## Abstract

**Background:**

Although human embryonic stem cells (hESCs) hold great promise as a source of differentiated cells to treat several human diseases, many obstacles still need to be surmounted before this can become a reality. First among these, a robust chemically-defined system to expand hESCs in culture is still unavailable despite recent advances in the understanding of factors controlling hESC self-renewal.

**Methodology/Principal Findings:**

In this study, we attempted to find new molecules that stimulate long term hESC self-renewal. In order to do this, we started from the observation that a commercially available serum replacement product has a strong positive effect on the expansion of undifferentiated hESCs when added to a previously reported chemically-defined medium. Subsequent experiments demonstrated that the active ingredient within the serum replacement is lipid-rich albumin. Furthermore, we show that this activity is trypsin-resistant, strongly suggesting that lipids and not albumin are responsible for the effect. Consistent with this, lipid-poor albumin shows no detectable activity. Finally, we identified the major lipids bound to the lipid-rich albumin and tested several lipid candidates for the effect.

**Conclusions/Significance:**

Our discovery of the role played by albumin-associated lipids in stimulating hESC self-renewal constitutes a significant advance in the knowledge of how hESC pluripotency is maintained by extracellular factors and has important applications in the development of increasingly chemically defined hESC culture systems.

## Introduction

Human embryonic stem cells (hESCs) are pluripotent cells derived from the inner cell mass of human blastocysts. The ability of hESCs to differentiate into any cell type of the adult human body, i.e. their pluripotency, has generated hopes that in the future these cells will be used as a source of differentiated cells to treat a number of human ailments, including type I diabetes, Parkinson's and heart disease [1]–[5]. However, before the potential of hESCs for regenerative medicine can be realized, many obstacles must be overcome. In this respect, one of the first issues that need to be addressed is the necessity of expanding hESCs in vitro in chemically-defined and animal product-free conditions [6]–[9]. Although significant advances are being made in this field, available media for hESC culture are still unsatisfactory due either to their undefined composition or, when composition is known, to their suboptimal performance [6], [8].

In order to develop chemically-defined media that robustly sustain hESC self-renewal, it is of critical importance that the signals and mechanisms controlling hESC fate choices (such as choosing to differentiate into a particular lineage as opposed to remaining undifferentiated) be understood in detail. Over the last few years, a number of extracellular factors have been identified that affect hESC self-renewal. Amongst these, a major role is played by members of the TGFβ superfamily of signaling molecules [10]–[16]. More precisely, TGFβ/Activin/Nodal stimulate hESC self-renewal by inducing phosphorylation of the intracellular mediators Smad2 and/or Smad3 [10]. On the other hand, BMPs act via phosphorylation of Smad1/5/8 to induce hESC differentiation to extraembryonic lineages [17], [18] or to germ cells [19]. Another important regulator of hESC self-renewal is bFGF, which seems to act in part by inducing the expression of TGFβ family molecules [20]–[22]. More controversial is the role of the Wnt family of proteins, for which apparently contradictory reports exist concerning their role in hESC maintenance [23], [24]. Most recently, IGF-II has also been described as a critical regulator of hESC self-renewal. IGF-II would be acting downstream of bFGF and in parallel with Activin/Nodal/TGFβ in order to maintain hESCs in a pluripotent state [20]. Another recent study has also confirmed the importance of IGF signaling for hESC self-renewal, as well as a role for Heregulin-1β, an ErbB2/3 ligand [25]. Finally, sphingosine-1-phosphate and PDGF have been shown to synergistically stimulate hESC self-renewal [26], whereas some neurotrophins, sphingosine-1-phosphate and a Rho-associated kinase (ROCK) inhibitor regulate human embryonic stem cell survival [27]–[29].

In this study, we find that albumin-associated lipids have strong positive effects on the self-renewal of two different hESC lines. Our results demonstrate that these lipids constitute an essential ingredient within knockout serum replacement, a supplement which is widely used to prevent hESC differentiation and stimulate hESC self-renewal.

## Materials and Methods

### Human embryonic stem cell culture

Human embryonic stem cell lines HUES7 and HUES9 were cultured on matrigel-coated plates. For plate preparation, BD Matrigel (BD Biosciences) was diluted 15 times in KO-DMEM (Invitrogen) and used to coat plates at 0.11 ml/cm^2^. Plates were incubated for 30 minutes at 37°C for matrigel to solidify and then washed once in D-PBS (Invitrogen) before being used. To maintain cell lines, these were cultured in mouse embryonic fibroblast-conditioned medium (MEFCM), which was prepared as previously described [30]. Briefly, hESC medium containing 20% knockout serum replacement (KOSR) and 10 ng/ml bFGF was incubated for 24 hours in the presence of mouse embryonic fibroblasts (MEFs), after which an additional 5 ng/ml bFGF was added to the medium before using it for hESC culture. For passaging, cells were incubated for 15 minutes at 37°C in 2 mg/ml dispase (Invitrogen) dissolved in D-PBS (1 ml dispase solution per well of a 6-well plate). After that, dispase was diluted by adding 4 volumes of KO-DMEM to the plate and cell clumps were raised by pipetting with a 5 ml tip (smaller tips cause excessive cell dissociation and subsequent differentiation) and transferred to 15 ml tubes. Cells were then centrifuged for 5 minutes at 400 g and the resulting pellet washed once more with 5 ml KO-DMEM. Finally, cells were resuspended in the desired medium and plated.

### Embryoid body differentiation

Embryoid bodies (EBs) were prepared by treating cells with 2 mg/ml dispase for 15 minutes at 37°C, as done when passaging them (see above). Cells were then fully detached by pipetting with a 5 ml tip, centrifuged, resuspended in DMEM+10% FBS and plated onto non-coated, non-cell culture-treated petri dishes. Despite this, some EBs still tended to attach to the plate, so every day the attached EBs were detached by gentle scraping with a sterile cell scraper. After 6 days of growth in suspension with media changes on days 2 and 4, EBs were transferred to gelatin-coated plates (0.1% gelatin for 2 hours at 37°C) in the same medium (DMEM+10% FBS). Under these conditions, cells attached to the bottom of the plates, where they were left to differentiate for 20 additional days, changing the medium as required.

### High performance liquid chromatography (HPLC)

2.5 ml of 50 mg/ml AlbuMAX II (Invitrogen) dissolved in PBS were extracted using a 1∶1 chloroform∶methanol mix (5 ml) and twice again with chloroform (2 ml each). The extracted lipids were then diluted to appropriate concentrations using chloroform∶methanol (1∶1) and used for HPLC analysis. HPLC standards consisted of 1 mg/ml each of triolein, cholesterol, ceramide, oleic acid, dioleoyl-phosphatidylglycerol, dioleoyl-phosphatidylcholine, dioleoyl-phosphatidylethanolamine, dioleoyl-phosphatidylserine, dioleoyl-phosphatidic acid, soy phosphatidylinositol, sphingomyelin and 0.5 mg/ml each of myristoyl-ceramide, oleoyl-lysophosphatidylcholine, oleoyl-lysophosphatidylethanolamine, oleoyl-lysophosphatidylglycerol and oleoyl-lysophosphatidylserine. The standards and the samples were injected into a normal phase HPLC column and analyzed using an evaporative light scattering detector (HPLC-ELSD).

### Flow Cytometry

For flow cytometric analysis, cells were washed once in D-PBS, incubated for 20 minutes at 37°C in cell dissociation buffer (Invitrogen) and individualized by pipetting. Cells were then centrifuged for 5 minutes at 400 g, resuspended in D-PBS, recentrifuged and resuspended again in blocking solution (BS, containing D-PBS+3% normal donkey serum), in which they were incubated for 5 minutes at room temperature. Next, cells were centrifuged once more and resuspended in BS with the desired antibodies (mouse anti-TRA-1-60 was diluted 100 times, as was mouse anti-cardiac myosin heavy chain, which was used as a negative control (both antibodies were from Chemicon)). After incubating cells for 30 minutes on ice with the primary antibodies, cells were washed three times in BS, incubated with secondary antibodies (Jackson Labs) as previously done for primaries, washed three more times in BS, filtered to remove any remaining cell aggregates and analyzed using a MoFlo flow cytometer (Cytomation). In case an analysis could not be performed immediately, cells were fixed in PBS+3% paraformaldehyde for 15 minutes at 4°C, washed twice in PBS, stored at 4°C in the dark and analyzed the next day.

### Karyotype analysis

Exponentially growing hESCs were treated overnight with 200 ng/ml KaryoMAX colcemid solution (Invitrogen) to arrest cells in metaphase. Next, cell medium was aspirated and cells were detached by a 20 minute incubation with cell dissociation buffer (Invitrogen). After two washes in PBS, cells were gently resuspended in a 0.56% KCl solution and incubated at room temperature for 15 minutes. Cells were then centrifuged and gently resuspended in cold methanol∶acetic acid 3∶1 (V∶V) for fixation. After incubating for 10 minutes on ice, cells were centrifuged again and resuspended in a small volume (about 200 µl per million cells) of the same cold methanol∶acetic acid solution. At this point, cells were dropped onto slides (approx. 10 µl/slide), slides were allowed to dry and then stained with KaryoMAX Giemsa stain (Invitrogen) for at least 2 hours. After washing the slides a few times with water, they were dried and visualized in a microscope for chromosome counting.

### Quantitative RT-PCR

Total RNA was purified from cells using Qiagen's RNeasy kit. The RNA concentration in the samples was determined spectrophotometrically and 1 µg of RNA per sample was converted to cDNA using Superscript II reverse transcriptase from Invitrogen. PCR reactions were prepared in microamp optical 96-well reaction plates (Applied Biosystems) by mixing 0.5 µl cDNA (1/40th of total), 5 µl SYBR green PCR master mix 2X (Applied Biosystems) and 10 picomoles of each primer in a total volume of 10 µl. Reactions were run in an ABI Prism 7900 Sequence Detector (Applied Biosystems) and results analyzed with SDS2.3 software (Applied Biosystems) using the ΔΔCt method. Primers used were hGAPDH-fwd (5′-GGA CTC ATG ACC ACA GTC CAT GCC-3′), hGAPDH-rev (5′-TCA GGG ATG ACC TTG CCC ACA G-3′), hOct4-fwd (5′-GAG AAG GAT GTG GTC CGA GTG TG-3′), hOct4-rev (5′-CAG AGG AAA GGA CAC TGG TCC C-3′), hNanog-fwd (5′-TGA ACC TCA GCT ACA AAC AGG TG-3′), hNanog-rev (5′-AAC TGC ATG CAG GAC TGC AGA G-3′), h-cTnT-fwd (5′-AGG CGC TGA TTG AGG CTC AC-3′), h-cTnT-rev (5′-ATA GAT GCT CTG CCA CAG C-3′), hGFAP-fwd (5′-CGG CTG CTT TTC CCT AAG C-3′), hGFAP-rev (5′-GGG TAC ATT TTG TGT GTG AGT AAG AAG-3′), hSox1-fwd (5′-GCC CTG AGC CGA CTG TGA-3′), hSox1-rev (5′-CCG TGA ATA CGA TGA GTG TTA CCT-3′), hSox17-fwd (5′-TGA ATG TGT CCC AAA ACA GCT T-3′), hSox17-rev (5′-CAC ACC CAG GAC AAC ATT TCT TT-3′).

### Immunocytochemistry

For immunofluorescence staining, cells grown on glass coverslips were fixed for 20 minutes at room temperature in PBS+4% paraformaldehyde followed by three washes in PBS. Next, cells were blocked and permeabilized by a 20-minute incubation with PBS-BS (PBS+0.5% BSA+0.05% saponin) and then incubated with mouse anti-TRA-1-60 antibody (Chemicon, diluted 1/100 in PBS-B (PBS+0.5% BSA)) for 1 hour at 30–37°C. After three washes with PBS-BS, cells were incubated for an additional hour at room temperature and in darkness with PBS-B containing 10 µg/ml DAPI or propidium iodide (Molecular Probes) and 0.5 µg/ml FITC-conjugated donkey anti-mouse antibody (Jackson Labs). Then, cells were washed five times with PBS and coverslips mounted on slides using Vectashield (Vector Laboratories). Slides were left overnight at 4°C and analyzed the next day by fluorescence microscopy.

### Alkaline Phosphatase staining

To stain cells positive for alkaline phosphatase, cells were first fixed by incubating them for 20 minutes at room temperature in 4% paraformaldehyde in PBS. Next, cells were washed three times with PBS and then three more times in NTMT buffer (100 mM Tris·HCl pH 9.5, 100 mM NaCl, 50 mM MgCl_2_ and 0.1% Tween-20). After this, cells were stained by incubating them at room temperature for 15–30 minutes in the dark in staining solution containing NTMT, 0.34 mg/ml NBT (Nitro-blue tetrazolium chloride, stock 75 mg/ml in 70% dimethylformamide) and 0.18 mg/ml BCIP (5-bromo-4-chloro-3′-indolylphosphate p-toluidine, stock 50 mg/ml in 100% dimethylformamide). Finally, the reaction was stopped by washing cells once in PBS and, after counterstaining with propidium iodide, cells were visualized in a microscope.

## Results

### Knockout serum replacement stimulates hESC self-renewal

**Figure 1:**
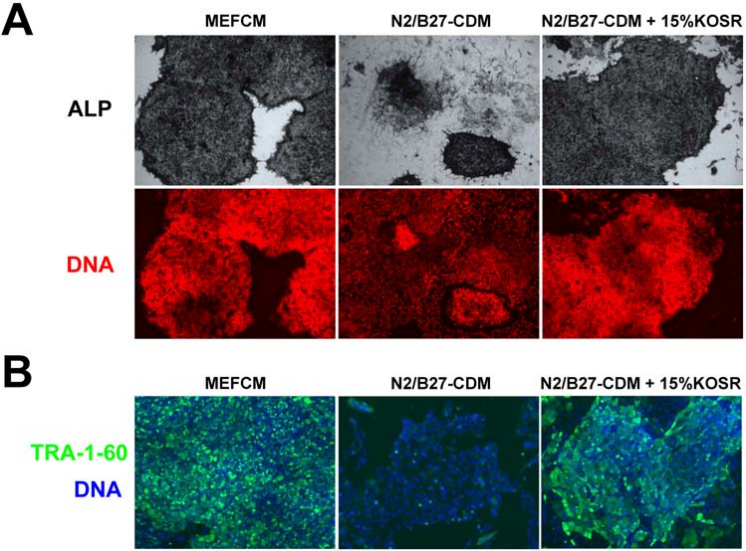
Knockout serum replacement (KOSR) stimulates hESC self-renewal. (A) HUES9 cells cultured for five passages in the indicated media were fixed and stained for alkaline phosphatase (ALP) activity (top row) or stained with propidium iodide to visualize DNA (bottom panel). (B) HUES7 cells cultured for five passages in the same media as above were stained with an anti-TRA-1-60 antibody (green) and with DAPI to visualize DNA (blue). Note how the majority of cells cultured in N2/B27-CDM are negative for the pluripotency markers ALP and TRA-1-60 whereas practically all cells cultured in N2/B27-CDM+15% KOSR or MEFCM are positive for these markers.

In order to find a chemically-defined medium (CDM) that robustly sustains long term hESC self-renewal, we started by testing those media that had already been reported to achieve this goal [14], [31]–[35]. However, in our hands none of these media was found to sustain hESC self-renewal in a satisfactory manner, that is, a manner comparable to what is achieved by culturing these cells on feeder layers or with feeder cell-conditioned medium (data not shown). Thus, in order to develop a better CDM for hESC culture, we decided to select one of these published media and work on its optimization. Our medium of choice, N2/B27-CDM [33], had the benefits of being inexpensive, easy to prepare and had given promising results in our preliminary tests. Nevertheless, our initial attempts to improve the performance of N2/B27-CDM by supplementing it with a variety of additional molecules (mostly chosen from the other above-cited published media) were to no avail. In one case, however, we did find a clear improvement in hESC self-renewal. This was achieved when HUES7 or HUES9 cells [36] were cultured in N2/B27-CDM supplemented with 15% knockout serum replacement (KOSR, Invitrogen). As can be seen in Figure 1, the proportion of HUES7/9 cells positive for undifferentiated hESC markers such as alkaline phosphatase (ALP) and TRA-1-60 was significantly greater when these cells were cultured in N2/B27-CDM+15% KOSR, as compared to culturing them in N2/B27-CDM alone. In particular, only a small percentage of cells grown in N2/B27-CDM for five or more passages maintained expression of ALP. These cells formed very small ALP^+^ clusters that were surrounded by much larger numbers of ALP^−^ differentiated cells (Figure 1A, central panels). By contrast, cells grown in N2/B27-CDM+15% KOSR were in their vast majority ALP^+^ and the colonies, in addition to being larger than those obtained without KOSR, were rarely surrounded by any ALP^−^ differentiated cells (Figure 1A, right panels). On the other hand, cells cultured with mouse embryonic fibroblast-conditioned medium (MEFCM), which also contains KOSR, grew even better and differentiated even less than the ones cultured in N2/B27-CDM+15% KOSR (Figure 1A, left panels). Analysis of TRA-1-60 protein levels by immunostaining confirmed the positive effect of KOSR in preventing hESC differentiation and favoring hESC expansion (Figure 1B).

### KOSR effects reside in its high molecular weight fraction

**Figure 2:**
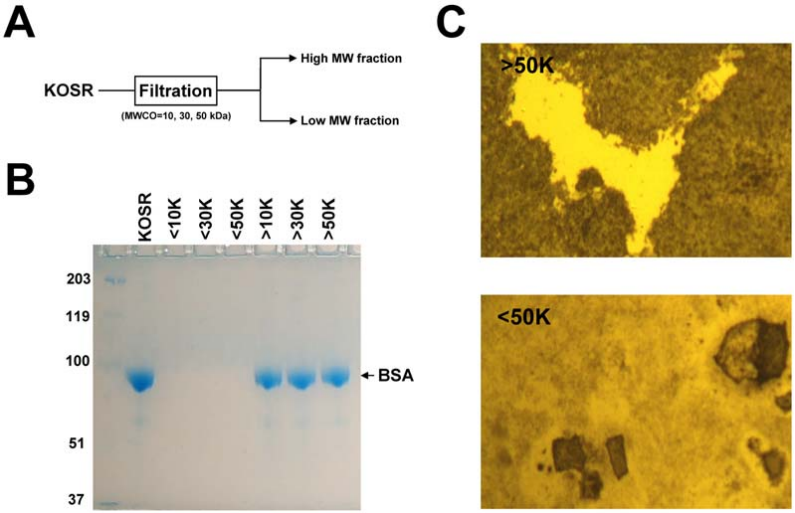
The active ingredient/s within KOSR reside in its high molecular weight fraction. (A) Samples of KOSR were filtered using Amicon Ultra filters (Millipore) of three different molecular weight cutoffs (MWCO), so that only molecules with molecular weights (MW) below the filters' MWCOs could go through them. For each filter, two fractions were obtained corresponding to the low and high MW fractions. The latter fractions were obtained by resuspending the molecules retained by the filters in a volume of D-PBS equal to the original KOSR volume. (B) The six fractions obtained in this manner were analyzed by SDS-PAGE to ensure that filters were working properly. Coomassie blue staining of the gel revealed that filters were indeed preventing high MW components to go through them. Lipid-rich BSA is the most abundant protein component in KOSR by a factor of about 1000 times and this is why no other proteins are seen in the gel. (C) HUES7 cells were cultured for five passages in N2/B27-CDM+15% (V∶V) of each of the six fractions. After this period of time cells were fixed and stained for ALP activity. As seen in the top panel for the >50 kDa fraction, all high MW fractions were able to maintain widespread ALP expression whereas, as shown in the bottom panel for the <50 kDa fraction, all low MW fractions failed to maintain hESC pluripotency, as assessed by ALP expression.

Since KOSR is not a chemically-defined product, the next question we attempted to answer concerned the identity of the molecule/s that are responsible for the effect of KOSR on the maintenance of hESC stemness. KOSR was originally designed and patented in 1998 by Life Technologies Inc. (now Gibco, part of Invitrogen) to substitute for fetal bovine serum (FBS) in applications, such as the derivation and culture of mouse embryonic stem cells, where the use of FBS was problematic due to its high lot-to-lot variability. Unlike FBS, KOSR has a very constant composition from one lot to another, thus eliminating the need to test every batch before being able to use it. The composition of KOSR can be found in its patent [37] and is shown in Table 1. In essence, KOSR is a mixture of small organic molecules (amino acids, vitamins and antioxidants), trace elements and three proteins, namely insulin, transferrin and lipid-rich albumin. Of these, the lipids associated to albumin are the only non-chemically-defined component of KOSR. In order to find out whether the active ingredient/s within KOSR are large or small molecules, we passed samples of KOSR through Amicon Ultra filters (Millipore) with molecular weight cutoffs (MWCO) of 10, 30 and 50 kDa (Figure 2A). As a result of this, two fractions were obtained for each filter, one containing large (above MWCO) and the other small (below MWCO) molecules, respectively. The integrity of the filters was confirmed by SDS-PAGE and found to be satisfactory (Figure 2B). Testing of all fractions on hESCs demonstrated that, whereas all the large molecular weight fractions were equivalent to KOSR in their ability to sustain hESC self-renewal, the low molecular weight fractions did not noticeably improve the results obtained with N2/B27-CDM alone (Figure 2C). From these data, we concluded that the active component of KOSR is a molecule of more than 50 kDa, which rules out all KOSR components except transferrin (77 kDa) and lipid-rich albumin (69 kDa for the albumin apoprotein).

**Table 1:** Composition of Knockout Serum Replacement

KOSR ingredients	
Amino Acids	Glycine, L-histidine, L-isoleucine, L-methionine, L-phenylalanine, L-proline, L-hydroxyproline, L-serine, L-threonine, L-tryptophan, L-tyrosine, L-valine
Vitamins/Antioxidants	Thiamine, reduced glutathione, ascorbic acid 2-PO_4_
Trace Elements	Ag^+^, Al3^+^, Ba^2+^, Cd^2+^, Co^2+^, Cr^3+^, Ge^4+^, Se^4+^, Br^−^, I^−^, F^−^, Mn^2+^, Si^4+^, V^5+^, Mo^6+^, Ni^2+^, Rb^+^, Sn^2+^, Zr^4+^
Proteins	Transferrin (iron-saturated), insulin, lipid-rich albumin (AlbuMAX)

### Lipid-rich albumin is responsible for the effects of KOSR

**Figure 3:**
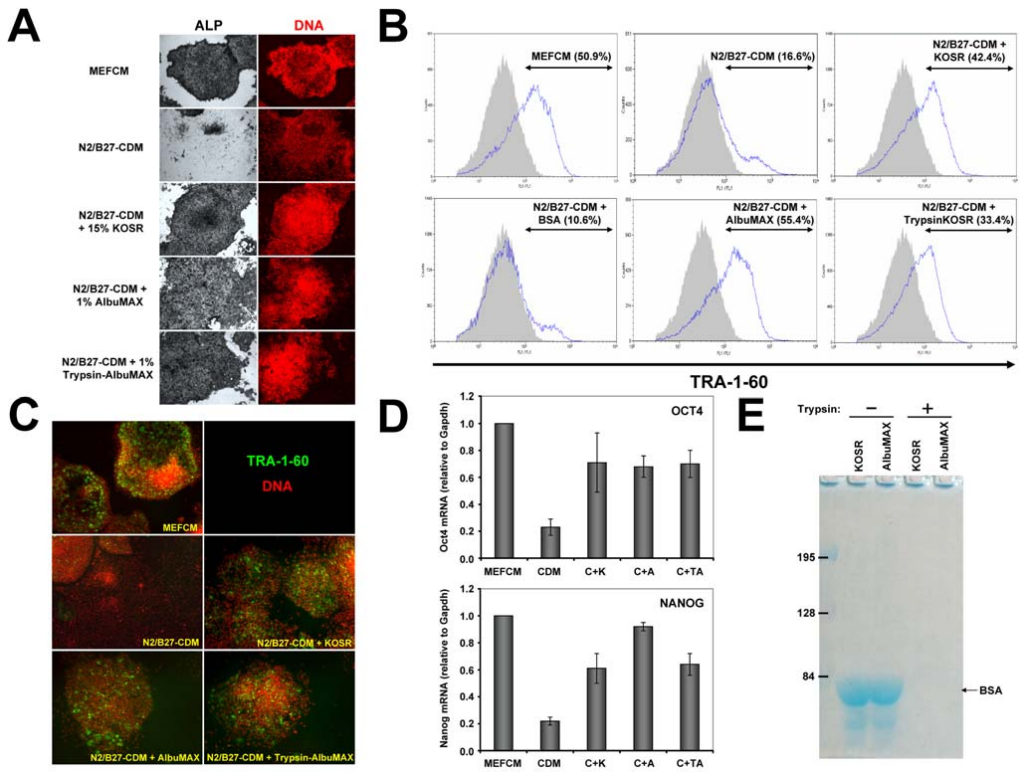
Lipid-rich BSA (AlbuMAX) is responsible for the effect of KOSR on hESC self-renewal. (A) HUES9 cells were cultured for five passages with the indicated media and at the end of this period they were fixed and stained for ALP activity and for DNA with propidium iodide. Note how the vast majority of cells cultured with N2/B27-CDM alone have lost ALP expression whereas cells cultured in the presence of KOSR, AlbuMAX or even trypsinized AlbuMAX still maintain high levels of ALP activity. (B) HUES7 cells were cultured in the presence of the indicated media for five passages and subsequently used to analyze expression of TRA-1-60 by flow cytometry. Note how TRA-1-60 expression has been lost in most of the cells cultured with N2/B27-CDM alone or with N2/B27-CDM+BSA (lipid-poor), whereas TRA-1-60 expression remains much higher in the presence of lipid-rich BSA (AlbuMAX) or KOSR, even after trypsinization of the latter. Percentages shown correspond to the % of cells in each sample (blue curves) giving a fluorescence signal higher than 10^2^ minus the % of cells giving this signal in the negative control (in grey, 5.8% of cells above 10^2^). (C) HUES9 cells were cultured for five passages in the indicated media, fixed and stained for TRA-1-60 expression (green) and with propidium iodide for DNA (red). Again, TRA-1-60 expression is lost in cells cultured with N2/B27-CDM alone but not when this medium is supplemented with KOSR, AlbuMAX or trypsinized AlbuMAX. (D) HUES9 cells cultured in the indicated media for five passages were used to analyze Oct4 and Nanog mRNA expression by quantitative RT-PCR. As before, supplementation with KOSR, AlbuMAX or trypsinized AlbuMAX significantly reduced the loss of Oct4 and Nanog that occurs with N2/B27-CDM alone as compared with MEFCM. Data are shown as mean +/− standard error of the mean (SEM) of two completely independent experiments. (E) Trypsinization of KOSR and AlbuMAX leads to the disappearance of BSA and other protein bands from these fractions, as observed by SDS-PAGE and Coomassie blue staining

Since N2/B27-CDM already contains a fairly large amount of transferrin [33], it is unlikely that the effect of KOSR is due to this molecule. Therefore, we decided to test whether the lipid-rich bovine serum albumin (lipid-rich BSA) present in KOSR, known commercially as AlbuMAX (Invitrogen), is able to recapitulate the effect previously observed with KOSR. Since the concentration of AlbuMAX in the KOSR supplement is ∼8% (g: 100 ml) [37], the amount of AlbuMAX present in a medium with 15% KOSR (V∶V) is ∼1%, so we tested the effect of 1% AlbuMAX on hESC self-renewal. As expected, both HUES7 and HUES9 cells cultured in N2/B27-CDM+1% AlbuMAX grew as well as those cultured in N2/B27-CDM+15% KOSR and significantly better than those grown in N2/B27-CDM alone (Figure 3). Therefore, the KOSR effect can be fully recapitulated with lipid-rich BSA (AlbuMAX).

### Albumin-associated lipids are responsible for the effects of AlbuMAX

In order to establish whether the effects of lipid-rich BSA (AlbuMAX) on hESC self-renewal are dependent on its protein or its lipid components, or both, we followed two different approaches. First, we compared the effects of AlbuMAX with those of a lipid-poor albumin (BSA fraction V, Fisher Scientific). As shown in Figure 3B, while AlbuMAX clearly stimulated self-renewal of HUES7 cells, the same amount of lipid-poor BSA had no significant effect, suggesting that lipids are necessary for the activity of AlbuMAX. Next, we tested the hypothesis that lipids are not only necessary but also sufficient, in the absence of protein, to stimulate hESC self-renewal. To do this, we analyzed whether or not deproteinization of AlbuMAX or of KOSR abolishes their activity (Figures 3A–E). This, in turn, was achieved by incubating KOSR (or a 10% AlbuMAX solution) for 30–60 minutes at 37°C in the presence of 1,300 U/ml (0.1 mg/ml) of bovine pancreatic trypsin (Sigma). After this, trypsin was inhibited by adding 0.5 mg/ml of soybean trypsin inhibitor (roughly four molecules of inhibitor per trypsin molecule; the inhibitor works with a 1∶1 stoichiometry). The resulting mixtures (hereafter referred to as Trypsin-AlbuMAX or Trypsin-KOSR) were tested for effects on hESC self-renewal (Figure 3A–D). In order to confirm that trypsin had efficiently degraded all existing proteins, we ran an SDS-PAGE gel to compare KOSR and AlbuMAX before and after the procedure (Figure 3E). As expected, no protein can be seen in these samples after trypsinization (according to the PeptideCutter program (www.expasy.ch/tools), the 607 amino acid residue sequence of BSA is predicted to have 45 cleavage sites for trypsin with a 100% probability of cleavage, and 26 additional sites with a probability higher than 80%). After culturing HUES7 or HUES9 cells for five passages in the presence of N2/B27-CDM alone or supplemented with either 15% Trypsin-KOSR or 1% Trypsin-AlbuMAX, it became evident that the trypsinized samples keep at least part of the activity of the original untreated KOSR or AlbuMAX samples, as measured by maintenance of the expression of ALP (Figure 3A), TRA-1-60 (Figures 3B and 3C), Oct4 and Nanog (Figure 3D), indicating that lipids associated to BSA are responsible for the effect on hESC self-renewal.

### Cells cultured with AlbuMAX remain pluripotent

**Figure 4:**
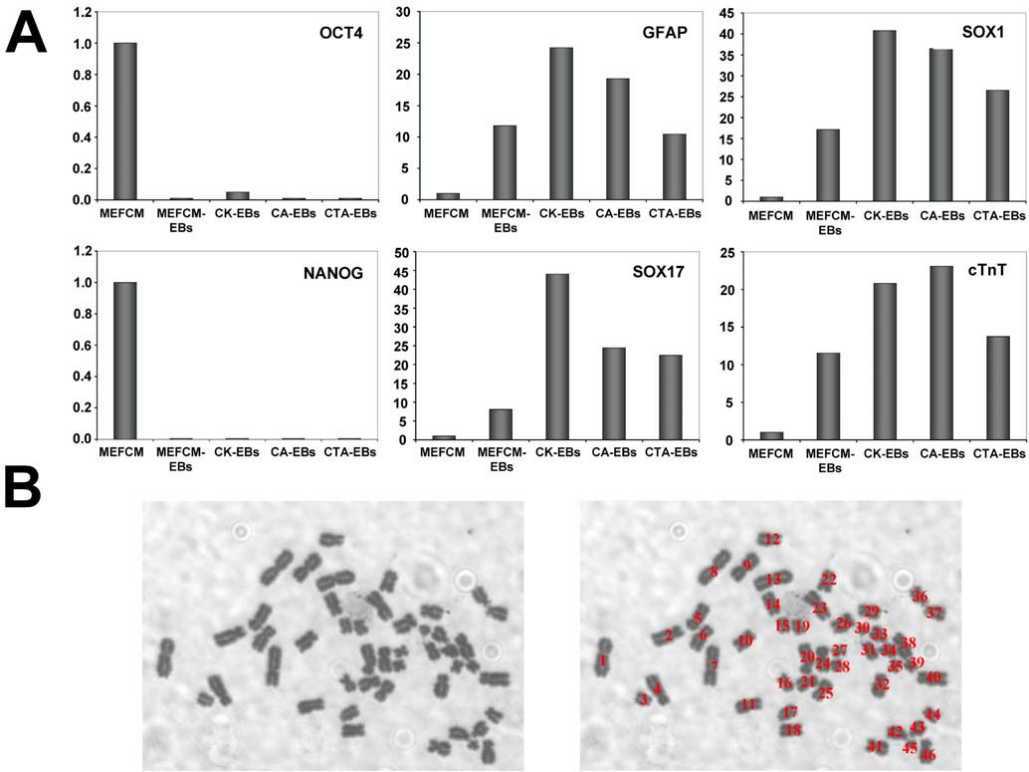
hESCs cultured with AlbuMAX maintain pluripotency and a normal karyotype. (A) HUES9 cells cultured for seven passages in the indicated media (MEFCM, N2/B27-CDM+15% KOSR (CK), N2/B27-CDM+1% AlbuMAX (CA) or N2/B27-CDM+1% Trypsin-AlbuMAX (CTA)) were used to generate embryoid bodies (EBs) as described in the Materials and Methods section. At the end of the EB formation and differentiation protocol (26 days in total), RNA was extracted from cells and used to measure the expression of different genes by quantitative RT-PCR. As a control, gene expression was also analyzed in undifferentiated HUES9 cells (cultured in MEFCM). As can be seen, differentiation occurred efficiently under all four conditions leading to an almost complete reduction in the expression of pluripotency markers (Oct4 and Nanog) as well as to very significant increases in the expression of ectoderm (GFAP and Sox1), endoderm (Sox17) and mesoderm (cTnT) markers. (B) Metaphase spread of HUES9 cells cultured for ten passages in N2/B27-CDM+1% AlbuMAX. The two pictures are the same, with the one below showing that there were 46 chromosomes in this cell.

Although it has been shown that hESCs cultured in N2/B27-CDM+AlbuMAX for several passages retain expression of previously described pluripotency markers such as ALP, TRA-1-60, Oct4 and Nanog, it remained to be proven that these cells are really pluripotent in the sense that they are capable of differentiating to all three germ layers of the embryo, i.e. ectoderm, endoderm and mesoderm. To address this issue, hESCs that had been cultured for seven passages in N2/B27-CDM plus AlbuMAX, KOSR or Trypsin-AlbuMAX (or MEFCM as a control) were induced to differentiate by formation of embryoid bodies (EBs) in suspension culture, as explained in the Materials and Methods section. After six days in suspension, EBs were transferred to gelatin-coated plates and cells allowed to differentiate further in adhesive conditions for 20 more days. After 10–15 days in adhesive culture, neurons and beating cardiomyocytes could be observed under the microscope under all conditions. At the end of the whole process, RNA was isolated from the different plates and the expression of several genes analyzed by quantitative RT-PCR (Figure 4A). As a control, gene expression in the differentiated cell populations was compared to that in undifferentiated cells (cultured in MEFCM). As can be seen in Figure 4A, differentiation under all four conditions led to a drastic reduction in the expression of pluripotency markers Oct4 and Nanog, while the expression of differentiated cell markers of ectoderm (GFAP and Sox1), endoderm (Sox17 and Pdx1 (the latter not shown)) and mesoderm (cardiac troponin T (cTnT)) was induced at least ten times in all cases. Therefore, hESCs that have been cultured with AlbuMAX for long periods of time retain their pluripotency.

### Cells cultured with AlbuMAX retain a normal karyotype

In addition to keeping the pluripotency of hESCs intact, it is important that media used to cultivate hESCs do not induce genomic alterations in these cells. To test whether culturing in the presence of N2/B27-CDM+AlbuMAX causes any changes in chromosome number, HUES9 cells were cultured for ten passages in this medium after which the number of chromosomes in the cells was counted as described in Materials and Methods. After these ten passages, 10 randomly picked metaphase spreads were counted and, of these, 9 had the expected 46 chromosomes (Figure 4B), and 1 had 47. Therefore, cells cultured in N2/B27-CDM+AlbuMAX for ten passages retain a normal number of chromosomes.

### Analysis of lipids contained in AlbuMAX

Since lipid molecules have an essential role in mediating the self-renewal enhancement observed with AlbuMAX, our next goal was to find out what lipid or lipids are responsible for this effect. In order to do this, we decided to take two different approaches. On the one hand, we carried out a lipid analysis aimed at identifying the major lipid species present in AlbuMAX. On the other hand, given that the active molecules in AlbuMAX need not be those that are more abundant and therefore appear more prominently in the lipid analysis, we also decided to test several lipids which we thought were good candidates to mediate the observed effect. This second approach will be dealt with in the next section. To identify lipids present in AlbuMAX, we extracted them using organic solvents and analyzed them by high performance liquid chromatography (see Materials and Methods section). The results of this analysis can be seen in Table 2. Lipids constitute approximately 0.65% of the dry weight of AlbuMAX. Of these lipids, more than 50% in weight corresponds to free fatty acids (∼54%), while the remainder is composed mainly of lysophosphatidylcholine (∼17% of all lipids), triacylglycerides (∼15%), phosphatidylcholine (∼8%), phosphatidic acid (∼3%), cholesterol and sphingomyelin (∼1% each).

**Table 2:** Lipids present in AlbuMAX

Lipid species	µg/ml in 1% AlbuMAX
Free fatty acids (FFAs)	35.29
Lysophosphatidylcholine (LPC)	11.30
Triacylglycerides (TAGs)	9.79
Phosphatidylcholine (PC)	5.29
Phosphatidic acid (PA)	2.07
Cholesterol (CH)	0.88
Sphingomyelin (SM)	0.80

### Testing of candidate lipids

**Figure 5:**
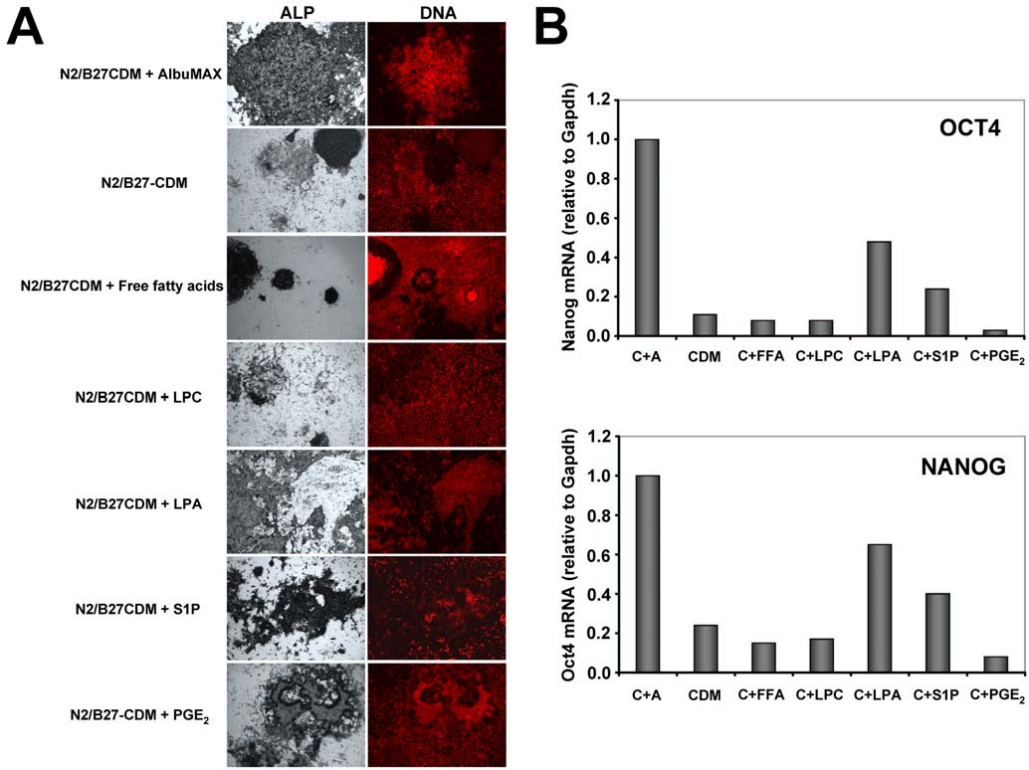
Testing of candidate lipids. HUES9 cells were grown in N2/B27-CDM alone, with 1% AlbuMAX or with the indicated lipids for six passages. At the end of this period cells were (A) stained for ALP activity and their DNA visualized with propidium iodide or (B) RNA was extracted from cells and the expression of Oct4 and Nanog mRNAs was analyzed by quantitative RT-PCR. Lipids used are: lysophosphatidylcholine (LPC), lysophosphatidic acid (LPA), sphingosine-1-phosphate (S1P), prostaglandin E2 (PGE2) and a chemically-defined mixture of saturated and unsaturated free fatty acids (FFA) known commercially as chemically defined lipid concentrate (Invitrogen).

Based on previous reports (see Discussion) and on the HPLC analysis just described, we decided to test the following lipids for effects on hESC self-renewal: lysophosphatidylcholine (LPC), lysophosphatidic acid (LPA), sphingosine-1-phosphate (S1P), prostaglandin E2 (PGE_2_) and a chemically defined mix of unsaturated and saturated free fatty acids (FFA) known commercially as chemically defined lipid concentrate (Invitrogen). To test these molecules, HUES9 cells adapted to grow on N2/B27-CDM+1% AlbuMAX were either maintained with this same medium or transferred to N2/B27-CDM alone or N2/B27-CDM supplemented with FFA (1/250 dilution), LPC (100 µM), LPA (10 µM), S1P (10 µM) or PGE_2_ (20 µM). Supplementation with 10 µM S1P led to massive cell death, so the S1P concentration was reduced to 3 µM, which was much less toxic for the cells. After six passages in the presence of the aforementioned lipids, cells were either fixed and stained for ALP (Figure 5A) or used to analyze Oct4 and Nanog mRNA expression by quantitative RT-PCR (Figure 5B). As expected, ALP, Oct4 and Nanog expression at the end of this experiment were much lower in cells cultured with N2/B27-CDM alone than in cells cultured in the presence of 1% AlbuMAX. On the other hand, cells cultured in the presence of FFA, LPC and PGE_2_ differentiated at least as much as cells cultured in N2/B27-CDM alone, indicating that none of these compounds prevents hESC differentiation. In addition, PGE_2_ seems to slow down cell division considerably, although this effect was not studied in detail. As for LPA and S1P, both lipids (especially LPA) seem to have a modest effect on hESC self-renewal, even though these effects, if they exist, are clearly weaker than the effect observed with AlbuMAX.

## Discussion

In this study, we have found that knockout serum replacement (KOSR) strongly stimulates self-renewal of human embryonic stem cells cultured with a previously reported chemically-defined medium (N2/B27-CDM) [33]. Since KOSR is not itself a chemically-defined product, we next identified the active ingredient within KOSR as lipid-rich BSA, also known as AlbuMAX. Therefore, AlbuMAX is the only ingredient of KOSR that is needed for hESC self-renewal and that is not already present in N2/B27-CDM. Furthermore, we have shown that both KOSR and AlbuMAX can be deproteinized by trypsinization and they still conserve a significant amount of activity, thus showing that albumin-associated lipids, rather than the albumin apoprotein, are responsible for the effect. Consistent with this, BSA that is not lipid-rich has no effect on hESC self-renewal. We have also established that hESCs cultured in N2/B27-CDM supplemented with AlbuMAX maintain their pluripotency and normal chromosome number after long-term passaging, indicating that this medium can be used for the expansion of hESCs in vitro.

The next question we have addressed concerns the identity of the active lipids in AlbuMAX. To this end, albumin-bound lipids were extracted with organic solvents and analyzed by high performance liquid chromatography (HPLC) (Table 2). Among the lipids identified by HPLC, some seem to be unlikely candidates for the effect being studied here, even if they cannot be ruled out completely. In particular, triacylglycerides are well known energy storage molecules but have no known function as signaling molecules that may affect cell fate [38]. Likewise, phosphatidylcholine, sphingomyelin and cholesterol are very ubiquitous plasma membrane lipids whose functions are mainly structural and as precursors for other, more active, lipid species [39]–[41]. Phosphatidic acid is a structural component of the plasma membrane as well, but it has recently been shown to be able to act as an intracellular second messenger in some contexts [42], [43]. On the other hand, free fatty acids (FFAs) have been shown to act as agonists for both nuclear receptors and G protein-coupled receptors (GPCRs) [44], [45], whereas lysophosphatidylcholine (LPC) is also capable of activating some GPCRs [46]. Therefore, since FFAs and LPC are the only identified lipids with known roles as extracellular signaling molecules, we decided to test their effects on hESC self-renewal, but no effects were observed. In the first case, given that there are many different FFAs and the HPLC was not able to resolve their individual identities, what we tested was a chemically-defined mix of saturated and unsaturated fatty acids (sold by Invitrogen as chemically-defined lipid concentrate). Even though the tested FFA mix had no activity (if anything it stimulated differentiation), it remains possible that the activity of AlbuMAX is due to one or more fatty acids that are not present among the ones tested, or even that the active one/s are in the mix but their activity is masked by others with antagonistic effects. In any event, since it seems unlikely that the active lipid/s in AlbuMAX are among those identified by HPLC and since it is perfectly possible that the levels of these active lipids are below the detection limit of our HPLC analysis, we also decided to test other lipids that, for different reasons, were good candidates for the effect. The lipids chosen were lysophosphatidic acid (LPA), sphingosine-1-phosphate (S1P) and prostaglandin E2 (PGE_2_). LPA and S1P are very active lysophospholipids that act by binding GPCRs on the cell surface, and many of these GPCRs have already been shown to be expressed by hESCs [26], [28], [46]. In the case of S1P, it has even been shown that this lipid stimulates self-renewal of hESCs cultured in the presence of feeder cells, whereas LPA was inactive under the same conditions [26], [28]. On the other hand, PGE_2_ was recently shown to stimulate self-renewal of hematopoietic stem cells [47], so we reasoned that it might also mediate similar effects on hESCs. However, when tested, not only did PGE_2_ not stimulate hESC self-renewal, it actually caused cell differentiation and a reduction in cell proliferation, thus ruling out PGE_2_ as a mediator of AlbuMAX's effects. Regarding S1P, high concentrations of this lipid (10 µM) induced a considerable amount of cell death, which is in stark contrast with a previous report showing that 10–20 µM S1P prevents hESC apoptosis [28]. In our opinion, the reason for this discrepancy probably lies in the presence of feeder cells in the previous study (no feeder cells were used here) or, alternatively, in the fact that different hESC lines were used in our (HUES7 and HUES9) and their experiments (Shef 1–6) [28]. In any case, we observed much less cell death when a lower S1P concentration (3 µM) was used. Under these conditions, S1P even seemed to have a slight positive effect on hESC self-renewal, but this effect was much weaker than that of AlbuMAX and therefore cannot account for it. Finally, 10 µM LPA also seemed to have a modest effect on hESC self-renewal but, again, this effect was weaker, as assessed by both morphology and marker expression, than that of AlbuMAX. Finally, other lipids that might in principle be considered candidates for the effect include steroid and other lipid hormones. Nevertheless, N2/B27-CDM already contains high amounts of progesterone, estradiol, thyroid hormone and corticosterone, so it is very unlikely that AlbuMAX effects are due to extra amounts of these molecules. Even so, we tested another glucocorticoid, dexamethasone, but it did not affect hESC self-renewal in our system (data not shown).

In conclusion, it is still unclear what lipid/s are responsible for the effect of AlbuMAX on hESC self-renewal. One possibility is that the active lipid is not among the ones tested here. Also possible is that the effect of AlbuMAX is due to a combination of lipids that must be added together. In this respect, the most promising combination on the basis of our results would be LPA+S1P, but this has not been tested yet. Likewise, it cannot be ruled out that some of the lipids tested here are active only at concentrations different from those used in our experiments (this is very unlikely in the case of LPC, which was used at 100 µM and whose concentration in AlbuMAX is about 25 µM (Table 2)).

In spite of not having been able to identify the lipid/s responsible for the effect of AlbuMAX, we think that the discovery of the role played by albumin-associated lipids in stimulating hESC self-renewal, together with the other results shown in this study, represent a significant advance in our knowledge of how hESC pluripotency is maintained by extracellular factors and has applications in the development of increasingly chemically defined hESC culture systems.
